# New insights in the immune treatment of Guillain–Barré syndrome

**DOI:** 10.1097/WCO.0000000000001408

**Published:** 2025-07-21

**Authors:** Eveline J.A. Wiegers, Bart C. Jacobs

**Affiliations:** a Department of Neurology; b Department of Immunology, Erasmus MC University Medical Center Rotterdam, The Netherlands

**Keywords:** clinical trials, comparative effectiveness research, complement, Guillain–Barré syndrome, immunoglobulin G, treatments

## Abstract

**Purpose of review:**

Guillain–Barré syndrome (GBS) is a severe but treatable form of immune-mediated neuropathy. The purpose of this review is to provide an update on current immune treatments for GBS, highlight challenges in clinical practice and research, and discuss new developments in therapies that focus on reducing inflammation and preventing further nerve damage.

**Recent findings:**

In 2023, a GRADE-based guideline was published on the diagnosis and treatment of GBS on behalf of EAN/PNS. Several clinical trials have been conducted in GBS recently, including studies with an observational comparative study design.

**Summary:**

Since 30 years, intravenous immunoglobulins and plasma exchange are the only proven effective immune treatments for GBS. Despite these treatments, a substantial proportion of patients recover incompletely and have residual disability or complaints with a high impact on quality of life. New treatment trials focus on reducing immunoglobulin G antibodies to nerves and inhibition of complement activation. Observational comparative studies based on extensive and well defined cohorts are an alternative method to evaluate the effect of treatments in GBS. Several novel study designs are discussed that aim to facilitate the conduct of future trials with more sustainable use of data.

## INTRODUCTION

Guillain–Barré syndrome (GBS) is the most frequent cause of acute flaccid paralysis with an incidence rate of 1–2 per 100 000 per year worldwide [1]. This immune-mediated polyradiculopathy has a typical monophasic disease course [2]. GBS usually starts with rapidly progressive weakness of the limbs, which may extend to cranial muscles (50%) and respiratory muscles (20%) [2,3]. In addition, patients with GBS may develop severe sensory deficits and autonomic dysfunction [4,5]. Following the progressive phase, which can last up to four weeks, patients stabilize and begin to improve over the course of month to years, although patients often recover incompletely [2].

Preceding infections are associated with GBS and include pathogens such as *Campylobacter jejuni*, *Mycoplasma pneumonia*, cytomegalovirus, Epstein–Barr virus, hepatitis virus, and Zika virus [6]. Infections with *C. jejuni* and *M. pneumoniae* trigger a cross-reactive antibody response to glycosylated antigens at peripheral nerve myelin and/or axon via molecular mimicry [2]. The pathogenesis of GBS following viral infections remains largely undetermined. Various patterns of antibody reactivity to nerve gangliosides and other glycolipids have been identified in GBS, correlating with clinical presentation and disease course [7^▪^]. Upon binding to nerves, these antibodies activate complement which results in dysfunction and injury of nerve axons and myelin [7^▪^]. Recent studies have also suggested the presence of nerve-targeting T-cells in GBS [8]. 

**Box 1:**
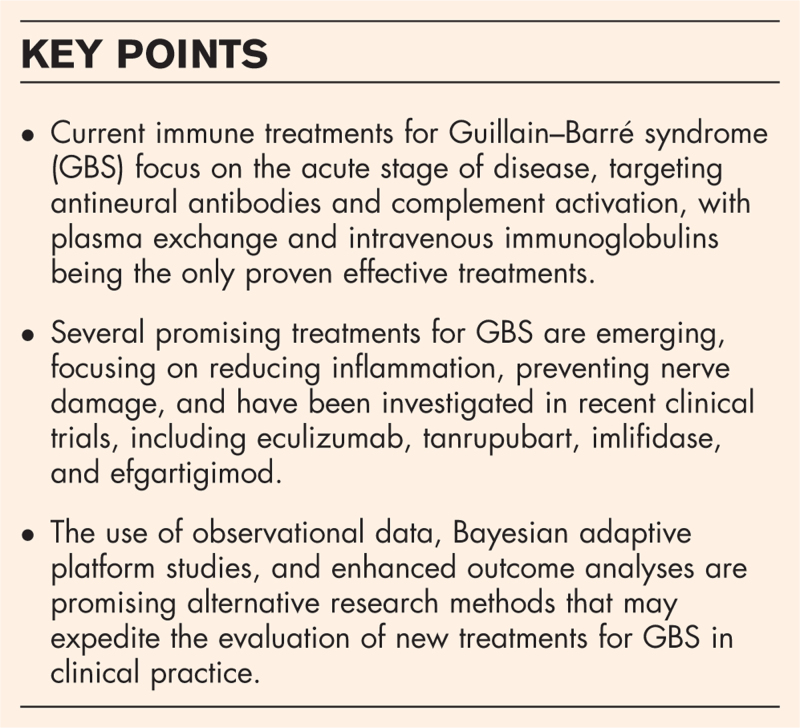
no caption available

The monophasic disease course of GBS is explained by the short-lasting immune response and the capacity of peripheral nerves to regenerate. Most patients are reaching clinical nadir within one week after onset of weakness, and at some stage of disease start to improve, even in the absence of treatment [2,5]. Preceding infections in GBS are usually self-limiting and frequently mild or subclinical. Serum antibodies targeting gangliosides typically disappear within a few weeks to months, however continued presence of these antibodies may occur and is associated with less favorable recovery [9]. All current immune treatments in GBS are focusing on the acute stage of disease, aiming to prevent further immune-mediated damaging of peripheral nerves. Specific targets of this treatment in GBS are antineural antibodies (neutralization or removal by plasma exchange, immune absorption, degrading enzymes, FcRn blockade, or saturation), complement activation (neutralizing C1q, C2, C3, and other complement factors), and their subsequent damaging effects (Table 1).

**Table 1:** Immune treatments in Guillain-Barré syndrome

Current available treatments					

Treatment	Mechanism of action	Treatment regimen	Indications	Start of treatment within	Outstanding questions
*Intravenous Immunoglobulins*[10^▪▪^]	*Pleiotropic immune modulatory effects*	*0.4 g/kg/day for 5 days*	*Unable to walk independently (GBS-DS ≥3), or rapid disease progression, swallowing difficulties, high risk of respiratory failure*	*2 weeks after onset of weakness*	*Treatment of milder forms, treatment-related fluctuations, timing of treatment, switch to PE in case of nonimprovement*
*Plasma Exchange*[10^▪▪^]	*Removal of antineural antibodies and other soluble immune mediators*	*4–5 exchanges over 1–2 weeks*	*Same as above*	*4 weeks after onset of weakness*	*Treatment of milder forms, treatment-related fluctuations, timing of treatment, switch to IVIg in case of nonimprovement*

Recent trials							

Treatment	Mechanism of action	Study design	Countries	Period	Patient numbers	Primary efficacy outcome	Key results
Eculizumab [17,18^▪^]	Humanized monoclonal antibody targeting C5	Phase II RCT, IVIg + eculizumab versus IVIg + placebo	Japan	2015–2016	34	Proportion able to walk at week 4	No difference in primary outcome, positive effect for some secondary outcomes.
		Phase III RCT, IVIg + eculizumab versus IVIG + placebo	Japan	2021–2022	57	Time to achieve ability to run	No clinical benefit
Tanruprubart [19^▪^]	Humanized recombinant monoclonal targeting C1q	Phase III RCT, 30 or 75 mg/kg versus placebo	Bangladesh, The Philippines	2021–2024	241	GBS-DS week 8	Clinical benefit of 30 mg/kg over 75 mg/kg and placebo.
Imflidase [21^▪^]	Enzyme targeting and cleaving IgG antibodies	Phase II, single-arm, imflidase + IVIg	France, The Netherlands, United Kingdom	2019–2024	30	Time to improve on GBS-DS, ability to walk	Median time to one-grade improvement: six days, to two-grade improvement: 16 days. Ability to walk: 67% at 8 weeks; 85% at 6 months

Ongoing trials							

Treatment	Mechanism of action	Study design	Countries	Period	Target number of patients	Outcome	Key results
Efgartigimod [24^▪^]	Human IgG fragment binding FcRn, enhances IgG degradation	Phase II, RCT, efgartigimod versus IVIg	United States of America	2023-ongoing	30	GBS-DS week 4	–

FcRn, neonatal fragment crystallizable receptor; g/kg, gram per kilogram; GBS-DS, Guillain–Barré syndrome disability score; IgG, immunoglobulin G; IVIg, intravenous immunoglobulins; PE, plasma exchange; RCT, randomized clinical trial.

The purpose of this review is to provide an update on current immune treatments for GBS, highlight challenges in clinical practice and research, and discuss new developments in therapies that focus on reducing inflammation and preventing further nerve damage. We analysed the literature from the last three years to identify the most recent studies related to GBS treatment. The search terms used in PubMed on April 14th, 2025, were: (“Guillain-Barre Syndrome”[MeSH] OR “GBS”[MeSH]) AND (“Therapy”[MeSH] OR “Treatment”[MeSH] OR “Drug Therapy”[MeSH] OR “Plasma Exchange”[MeSH] OR “Immunotherapy”[MeSH] OR “Intravenous Immunoglobulins”[MeSH] OR “Corticosteroids”[MeSH] OR “Immunoglobulins, Intravenous”[MeSH] OR “Treatment Outcome”[MeSH]) AND (“humans”[MeSH Terms] AND English[lang]). Additional clinical trials were consulted via clinical trial protocols.

## GUIDELINES FOR THE TREATMENT OF GUILLAIN–BARRÉ SYNDROME

Various guidelines have been published on the treatment of GBS. A consensus guideline to support clinicians in a global setting was developed in response to the Zika virus outbreak (2015–2016) [3]. In 2023, the European Association of Neurology and the Peripheral Nerve Society (EAN/PNS) published a GRADE-based guideline on the diagnosis and treatment of GBS [10^▪▪^]. According to the guideline, plasma exchange (PE) and intravenous immunoglobulins (IVIg) are the only proven effective immune treatments for the acute stage of GBS [10^▪▪^]. There is no evidence supporting the efficacy of corticosteroids or other immune treatments in GBS [11]. PE and IVIg are considered equally effective and the preference largely depends on local costs and availability. The recommended regimen for IVIg is 0.4 g/kg/day for five consecutive days, and for PE is 4–5 exchanges over 1–2 weeks (or 2 exchanges in mild forms of GBS) [10^▪▪^].

The decision to start immune treatment in GBS depends on clinical severity (measured by the GBS disability score, GBS-DS), rate of disease progression, and time from onset of weakness. In general, there is an indication to start treatment in patients who are unable to walk independently (GBS-DS ≥3), have rapid disease progression, develop swallowing difficulties, and/or are at high risk of needing ventilator support.

For milder forms of GBS, treatment could be considered within two weeks of onset, although a previous observational comparative study found no effect of IVIg compared with no treatment. However, some of these patients further deteriorate and many experience residual complaints [12]. To assess the effectiveness of a new treatment in preventing disease progression and residual symptoms, in patients with milder forms of GBS a study design where patients are randomized to receive either a new treatment or a placebo at the earliest stage of the disease could be considered.

## CONTROVERSIES AND CHALLENGES IN CURRENT TREATMENT STRATEGY

Despite the proven efficacy of IVIg in GBS, some patients show no clinical response after start of treatment or even further deteriorate. Previous studies have found that the pharmacokinetics after a standard course of IVIg are highly variable among patients with GBS, with rapid clearance being associated with poor recovery [13]. Administering a second dose of IVIg in patients with a poor predicted outcome does not improve recovery and increases the risk of complications, and is therefore not recommended [14]. A second course of the initial treatment is recommended only for treatment-related fluctuations occurring within 8 weeks of symptom onset, which affect 10% of patients [10^▪▪^]. At present, it is undefined if patients not responding to IVIg could benefit from switching to PE.

The EAN/PNS guideline recommend initiating treatment within 4 weeks after the onset of weakness and starting treatment as soon as possible in patients who are able to walk unaided [10^▪▪^]. A recent study indicated that treatment is started beyond seven days after onset in a quarter of the patients, suggesting room for improvement [15]. For various acute neurological diseases, earlier treatment has resulted in better outcomes [16]. A recent retrospective study involving over 100 IVIg-treated GBS patients admitted to Korean centers found that a shorter time to IVIg administration was independently associated with better outcomes up to 12 months after disease onset [15]. This finding supports the importance of early diagnosis and start of treatment in GBS. Further research is required on the variation in the duration of the active disease in GBS, which would provide a rationale for a more personalized treatment window.

## RECENT AND ONGOING CLINICAL TRIALS IN GUILLAIN–BARRÉ SYNDROME

Eculizumab, a humanized monoclonal IgG2/IgG4 antibody targeting C5, is designed to prevent complement activation, thereby limiting tissue damage. In a phase II RCT, Japanese patients with GBS who were unable to walk were randomized to receive IVIg plus either eculizumab (900 mg) or placebo. The results indicated that the primary endpoint of walking independently at week 4 was not met. However, patients receiving eculizumab experienced slightly better functional long-term outcomes as indicated by reaching the ability to run [17]. The efficacy of eculizumab as an add-on therapy to IVIg was further studied in a phase III trial involving 57 patients with GBS from Japan. Despite being well tolerated and significantly reducing serum C5 concentration, eculizumab did not demonstrate improvements in functional outcomes compared to the placebo [18^▪^]. Consequently, the EAN/PNS guideline does not recommend eculizumab as a treatment for GBS [10^▪▪^].

Tanruprubart is a fully humanized recombinant IgG4 monoclonal antibody designed to block C1q and the activation of the classical complement pathway. A phase III RCT investigating tanruprubart in patients with GBS has recently been completed in Bangladesh and the Philippines [19^▪^]. In both countries, many patients receive best supportive care only due to the unavailability or unaffordability of IVIg or PE. In total, 241 patients who were unable to walk independently and within 10 days of onset of weakness (and not treated with IVIg or PE) were randomized to receive a single intravenous infusion of either 30 mg/kg or 75 mg/kg tanruprubart, or placebo. It was observed that both doses inhibited complement activation, but compared to placebo and 75 mg/kg, patients receiving 30 mg/kg of tanruprubart demonstrated better neurological outcomes at week 8 and obtained independent mobility earlier [19^▪^].

Imlifidase, already approved for highly sensitized kidney transplant candidates, is an enzyme that specifically targets and cleaves IgG antibodies [20]. Recently, a single-arm phase II clinical trial studying the efficacy of imflidase in GBS was completed [21^▪^]. This study enrolled 30 patients unable to walk independently, presenting within 10 days of onset of weakness, and meeting the criteria for IVIg treatment according to local protocols. On day 1, patients received a dose of 0.25 mg/kg of imlifidase, followed by a full course of IVIg, starting at least 48 h after imlifidase administration. Patients were assessed for safety, adverse events, and functional outcomes. The results indicated that the treatment was safe and well tolerated. By the first week, the mean muscle strength had improved by 10.9 points on the Medical Research Council sum score compared to baseline. The median time to a one-grade improvement on the GBS-DS was 6 days, and 16 days for a two-grade improvement. By eight weeks, 67% of patients were able to walk independently, which increased to 85% at 6 months [21^▪^].

Efgartigimod, a human IgG antibody Fc fragment designed to target the neonatal Fc receptor, enhances IgG degradation and is being investigated as a potential therapeutic agent for GBS. A recent case report described the disease course of two Chinese patients with severe GBS who were treated with efgartigimod. Both patients exhibited rapid clinical improvement and favorable outcomes, with the treatment being well tolerated [22^▪^]. These findings are consistent with a recent phase II study in patients with Chronic Inflammatory Demyelinating Polyneuropathy, which demonstrated the efficacy of subcutaneous efgartigimod in reducing the risk of relapse compared to placebo [23]. A phase II clinical trial for GBS which evaluates the efficacy and safety of efgartigimod (20 mg/kg on days 1 and 5) compared to IVIg is currently in progress [24^▪^].

## IMPROVING EFFICIENCY OF TREATMENT STUDIES IN GUILLAIN–BARRÉ SYNDROME

Further studies are needed to understand the implications of new treatments for routine clinical practice. Although randomized controlled trials (RCTs) are the gold standard in providing evidence on treatment efficacy, RCTs are increasingly criticized for poor generalizability [25,26]. RCTs in GBS face several challenges, including timely and adequate enrollment of patients with this acute and rare disease. The conduct of RCTs in GBS is further complicated by the heterogeneity in the pathogenesis, clinical severity, and course of disease. Additionally, ethical concerns arise when potential treatments cannot be administered alongside the current standard of care (IVIg/PE). These issues highlight the need for more efficient trial strategies and encourage researchers to explore alternative study designs and more sustainable usage of previously acquired data.

### Use of observational datasets

An emerging approach in the field of rare diseases, involves leveraging observational data to estimate treatment effects. This can be achieved through two methods: utilizing observational data independently, and augmenting clinical trial data with observational data.

In the first option, high-quality observational datasets enable comparative effectiveness research (CER) by comparing outcomes between patients who received specific treatments and those who did not. CER measures differences in outcomes in daily clinical practice across diverse populations and is typically designed for already approved interventions [27]. The absence of randomization in observational studies increases the risk of confounding by indication, where reasons to administer treatment are linked to patient prognosis. To address this, methods like instrumental variable analysis and propensity scores are used. An example of CER is the observational study in which one versus two courses of IVIg in patients with a poor prognosis was compared in the cohort from the International GBS Outcome Study (IGOS). Using propensity score matching, the study found that patients receiving a second course of IVIg had worse outcomes than those receiving only one course [28]. Although these findings were later confirmed in a RCT [14], unobserved confounding factors likely influenced clinicians to administer a second course of IVIg. These factors, which could not be accounted for with propensity score matching, may have led to the observed results and limit the interpretation of these findings.

In the second option, observational data are used as an external control arm for comparison with patients from phase II or III trials. Two examples of this approach in GBS are the ongoing studies in which patients from IGOS cohort are compared to patients from the trials with imlifidase and tanruprubart. Patients from the single-arm trial investigating the safety and efficacy of imflidase followed by IVIg are compared with patients from IGOS treated with IVIg only meeting the inclusion criteria from the trial [29]. To compare the effectiveness of tanruprubart versus IVIg/PE a framework has been developed, in which the placebo arm will be replaced by patients in IGOS that match eligibility criteria for the original trial [30]. Propensity score methods can be used to establish comparable clinical profiles between patients in the intervention and external control arms, aiming to achieve unbiased efficacy estimates. It has been discussed that emulation of a RCT can be improved if an observational database closely matches the RCT design [31]. This might be particularly relevant when replacing the placebo arm of the phase III trial investigating the efficacy of tanruprubart, as identifying a sufficient number of patients treated with IVIg or PE in these countries will be challenging.

### Bayesian adaptive platform studies

Bayesian adaptive platform studies enhance trial efficiency by leveraging randomization and allowing simultaneous evaluation of multiple treatments under a single protocol. Participating centers need to meet basic requirements only once, saving time and resources in data collection, analysis, and ethical approval. Successful examples include REMAP-CAP [32] in pneumonia and TRICALS [33] in amyotrophic lateral sclerosis. Bayesian randomization updates the probability of hypotheses with new data and adaptive randomization adjusts patient allocation based on interim results [34]. This is also a promising design for accelerating the evaluation of new treatments for rare and complex diseases like GBS.

### Outcome analyses

Outcome analyses are a crucial component of investigating treatment effects. Most clinical trials in GBS use as primary endpoint the GBS-DS, an ordinal scale assessing functional status with scores from 0 (healthy) to 6 (death). In clinical trials it is common practice to dichotomize ordinal scale into categories, such as the ability to walk versus inability to walk for the GBS-DS, although this reduces the statistical power [35^▪^]. An alternative method to prevent this loss of information is an ordinal analysis in which the rank ordering of the full GBS-DS scale is used [35^▪^].

GBS trials typically include repeated outcome measurements over time, allowing for longitudinal analyses to characterize how treatment efficacy evolves throughout the study period. This approach is particularly valuable in monophasic diseases like GBS, where temporal variations in response may otherwise go unnoticed. The application of longitudinal methods remains uncommon in GBS trials, highlighting a potential area for methodological improvement [35^▪^].

While widely used to evaluate treatment effectiveness, the GBS-DS primarily focuses on disability in walking and respiration and does not capture other effects of GBS, including arm functioning, pain, psychological well being, and quality of life. This has led to increased interest in patient-reported outcome measures (PROMs), which are standardized instruments for patients to report health status and treatment outcomes [36]. However, PROMs are criticized for being subjective and prone to bias. Blinded RCTs with placebo control and collecting multiple PROMs over time in combination with standardized clinical outcome measures are suggested to improve evidence of efficacy [36].

## CONCLUSION

In summary, several promising treatments are emerging for GBS, focusing on reducing inflammation and preventing further nerve damage. Various challenges in GBS research complicate the conduct of RCTs. Alternative research methods may be employed to expedite the evaluation of these treatments’ applicability to daily clinical practice.

## Acknowledgements

*The authors acknowledge the use of Microsoft Copilot in drafting this manuscript to refine language and assist with editing for clarity and coherence. To comply with all scientific and ethical standards, the authors critically reviewed and assessed all content to ensure accuracy and compliance.*

### Financial support and sponsorship

*None.*

### Conflicts of interest

*E.W. does not report any conflicts of interest. B.J. received support for research from Prinses Beatrix Spierfonds, GBS-CIDP Foundation International, Annexon, CSL-Behring, Grifols, Roche, Hansa Biopharma and Octapharma. B.J. is chairing the Steering Committee of the International GBS Outcome Study (IGOS).*

## REFERENCES AND RECOMMENDED READING

Papers of particular interest, published within the annual period of review, have been highlighted as:

▪ of special interest
▪▪ of outstanding interest
